# Domestic Cow-Related Severe Facial Trauma in an Older Farmer Undergoing Anticoagulation Treatment

**DOI:** 10.7759/cureus.29818

**Published:** 2022-10-01

**Authors:** Yoshihiro Yamamoto, Yoshihiro Aoki

**Affiliations:** 1 Department of Emergency and Critical Care Medicine, Aizawa Hospital, Matsumoto, JPN

**Keywords:** early intubation, mandible fracture, direct oral anticoagulants (doac), geriatric emergency medicine, oral anticoagulation, facial trauma, intensive treatment, human-animal conflict, cattle farmers, aging

## Abstract

Domestic cow-related injuries can carry significant morbidity and mortality among livestock farmers. We report a case of an 83-year-old male farmer with severe facial trauma and a potentially compromised airway caused by a domestic dairy cow during his routine work while taking oral anticoagulants. Since head and neck trauma caused by domestic cows can be severe, preventive measures should be taken to protect cattle farmers in those exposed areas.

## Introduction

Dairy cows are important animals in the livestock industry worldwide; however, many traumatic injuries due to dairy cows have been reported [1-3]. Most reported human injuries caused by domestic cows were blunt trauma to the trunk [3,4]. At the same time, trauma in older adult patients increases with age and physical inability [5]. Although the livestock industry is essential even in developed countries, both workforce and population aging are accelerating [6,7]. In this sense, increasing numbers of older farmers can suffer severe trauma from domestic dairy cows. Nevertheless, the risk details have rarely been explored.

Anticoagulation is an essential medication for cerebrovascular infarction and atrial fibrillation [8]. While protecting against further thromboembolic events, there is always a risk of bleeding. In the case of trauma, anticoagulation increases the risk of bleeding, especially traumatic brain injury [8]. Accordingly, older adults with these underlying diseases and under anticoagulation may be at high risk for severe injury. In this paper, we report a case of an older farmer undergoing anticoagulation treatment with severe facial trauma caused by a domestic dairy cow during his routine work.

## Case presentation

**Figure 1:**
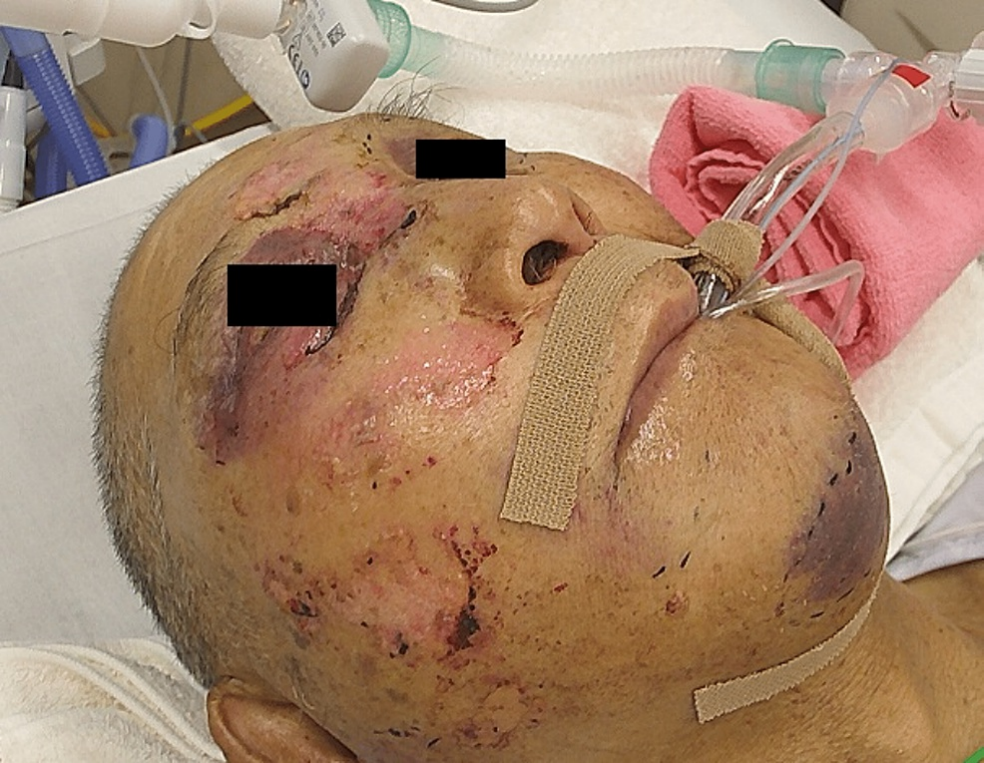
Facial appearance after intubation on admission day Prominent swelling from the right cheek to the mandible due to bilateral mandibular fractures is observed

An 83-year-old male with a history of atrial fibrillation, chronic heart failure, and chronic kidney disease was admitted to the emergency department with facial trauma and oral bleeding. He had been taking edoxaban for his underlying condition. The patient had been engaged in livestock farming for 50 years. On the day of his hospital visit, he had dropped his belt loop clip near a dairy cow over the fence. When he squatted down to retrieve it, the cow was immediately excited. The neighboring cow also became reactively excited and rushed at the patient, and its head hit the patient’s face. The patient’s right side of the face was severely bruised, and bleeding was observed in the mouth. On admission, following the airway, breathing, circulation, disability, and exposure (ABCDE) approach, the airway was maintained with speech, and tachypnea with a respiratory rate of 24/minute and normal oxygen saturation of 100% (room air) without abnormal chest auscultation were noted. The radial artery was well palpated with blood pressure of 142/82 mmHg, but mild irregular tachycardia with a pulse of 116/minute was observed. The Glasgow Coma Scale rating was 15 points, and disturbance of consciousness was not noted; body temperature was 37.7°C, and oral bleeding was observed. Physical examination revealed prominent swelling in the right cheek, abrasion from the right cheek to between the eyebrows, fractured teeth and venous bleeding from gingival laceration, and mild swelling of the floor of the mouth (Figure 1).

**Figure 2:**
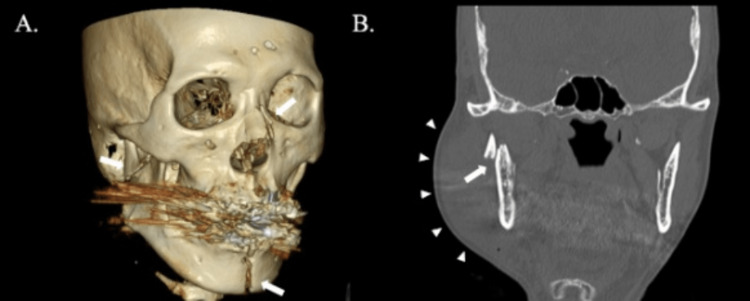
Facial computerized tomography (CT) on admission (A) Facial three-dimensional (3D) reformatted CT images on admission. Fractures of the left mandible body, right mandible head and neck, and nasal bone are observed (white arrows). (B) Coronal section of facial CT image on admission. Right mandible head fracture (white arrow) and soft tissue swelling are observed (white arrowheads)

A blood test performed on admission revealed hemoglobin of 10.3 g/dL (normal range: 13.7-16.8), platelet of cells of 25.0 × 10^4^/μL (normal range: 15.8-34.8), prothrombin (PT) time of 16.8 seconds (normal range: 9.8-12.1 seconds), PT-international normalized ratio of 1.38 (normal range: 0.88-1.09), and partial thromboplastin time of 30.1 seconds (normal range: 24.5-41 seconds). A computed tomography (CT) scan showed fractures of the bilateral mandible and nasal bone (Figure 2A-2B).

Hemostasis was achieved with intraoral gauze packing. However, the swelling of the floor of the mouth worsened over time. Therefore, prophylactic tracheal intubation was performed due to the possible risk of airway obstruction. He was admitted to the intensive care unit (ICU) for ventilator management. Edoxaban was ceased to avoid further progressive bleeding.

Enteral feeding was begun on the third day via a nasogastric tube. After the swelling improved, a right-sided mandibular hemiarthroplasty was performed on the eighth day. The fracture fragments were repaired and fixed with plates and locking screws. The three-hour surgery was completed with a drain tube inserted around the frontal neck area. A blood test on the eighth day revealed hemoglobin of 7.4 g/dL, so the patient received a transfusion of four units of red blood cells. The patient was extubated on the following day after ascertaining a systemically stable condition. Oral feeding was begun on the ninth day. Edoxaban was resumed orally on the 10th day of hospitalization. The drain tube was removed on the 13th day. Oral and cervical stitches were removed on the 15th day.

As a cognitive function test, the Mini-Mental State Examination (MMSE) was conducted on the 10th day. The patient’s initial score was 19 points, which improved to 27 on the 16th day. On the 20th day, he was transferred to the rehabilitation ward to continue long-term rehabilitation. Finally, the patient’s oral and swallowing functions were positively assessed, and he was discharged on the 50th day with no sequelae.

## Discussion

Various consequences of conflicts between humans and livestock farming animals have been reported. Some examples include the loss of productivity due to damage to infrastructure and time, the financial cost of farmer security, labor losses due to trauma, zoonotic diseases, and psychological stress [1]. The livestock that most commonly causes injuries to humans are cattle, horses, and sheep [1]. Cattle-related trauma includes fractures, sprains, bruises, blunt injuries, cuts, and organ damage [1,9].

Although the patient was a skilled livestock farmer, he sustained a severe facial trauma injury from a domestic dairy cow. In cattle-related trauma, bulls can cause injury by kicking, headbutting, and stomping [3]. The most frequent injuries by bulls are located in the lower extremities, perineum, and abdomen [10]. However, blunt trauma is common in dairy cows as the horns are usually removed soon after birth [11]. Organ, soft tissue, and vascular damage can also occur [6,10,11]. Considering the cattle’s body structure and size, a collision of its head and neck with a human’s abdomen and lower limbs can be the primary etiology. Therefore, although the head and neck area may not be expected parts of injury, several cases of cattle attacks while the victim’s sitting or working have been reported, same as in our case [3]. Although previous reports of cattle-related trauma indicate that severe cases may require intensive care, the mortality rate has been reported at approximately 1% [6]. However, though rare, head and neck injuries by cattle could be severe [6]. In addition, facial trauma may cause airway obstruction, which requires intensive care [12,13]. The present case was an exclusively facial injury, but our patient required intubation and ventilator management.

Anticoagulants include heparin, warfarin, and direct oral anticoagulants (DOACs). In recent years, warfarin has been replaced by DOACs in some cases because they do not require routine monitoring and have fewer bleeding complications [8]. However, the risk of bleeding has still been reported in DOAC use [14]. Therefore, when dealing with trauma, patients on DOACs should also be treated carefully. The elderly population on DOACs for atrial fibrillation is large and growing, and they often continue to perform physical work well beyond retirement age [15]. Facial trauma among this population warrants early airway protection by emergency physicians, considering airway being compromised due to progressive bleeding and swelling around the neck.

Fractures of the mandible are classified according to the fracture site as mental part, the body of the bone, mandibular angle, mandibular branch, muscular process, and articular process [16]. Surgical treatment is indicated in cases of mandibular fractures with deviated fracture fragments, open fractures, multilinear fractures, comminuted fractures, and atrophy of the edentulous jaw with the loss of bone mass and quality and in cases where there is concern that non-percutaneous treatment will not allow adequate recovery of form and function or bone fusion healing [17]. In addition, surgical intervention can allow the recovery of the oral and maxillofacial function, and the patients can return to social life significantly earlier than with nonoperative treatment [17,18]. In the present case, the functional outcome after the surgical treatment was also good.

Given the increase of injury in occupations involving cows, trauma prevention is essential [1]. It is necessary to manage animal factors in the human-livestock conflict. For example, cows are unable to recognize nearby objects immediately, so sudden squatting in front of them may excite them [1]. Therefore, bending down in front of a cow and approaching without warning should be avoided. In addition, wearing protective equipment is critical to prevent severe head trauma [3].

Our patient was usually careful to keep a substantial distance from cows, but due to the accident of dropping his items, he misjudged the situation and was unable to take appropriate action and avoid trauma. Although the patient did not have dementia, considering his age, declined physical function and impaired judgment ability associated with aging may have contributed to his severe injury. It was reported that trauma during agricultural work was more common in older males, and human factors in animal-related trauma may involve the patient’s cognitive and physical functional aspects [3,18]. Given the aging population of livestock farmers, similar cases should receive attention.

## Conclusions

We present a case of severe facial trauma caused by a dairy cow in an older farmer while taking oral anticoagulants. Facial trauma in people being treated with anticoagulation warrants early airway protection by emergency physicians. The risk of trauma in the livestock industry may increase due to declined physical and judgment functions associated with aging. In addition, since head and neck trauma by cattle can evolve severely, preventive measures to protect these areas should be taken, especially in the aged farmers.
